# Benzilic acid: a monoclinic polymorph

**DOI:** 10.1107/S2414314624010393

**Published:** 2024-10-31

**Authors:** Petrus Prinsloo, Eric Cyriel Hosten, Richard Betz

**Affiliations:** a Nelson Mandela University, Summerstrand Campus, Department of Chemistry, University Way, Summerstrand, PO Box 77000, Port Elizabeth, 6031, South Africa; Goethe-Universität Frankfurt, Germany

**Keywords:** crystal structure, polymorph, benzilic acid

## Abstract

The title compound, C_14_H_12_O_3_, is an α-hy­droxy­carb­oxy­lic acid whose ortho­rhom­bic polymorph has been reported earlier [Qiu *et al.* (2007 ▸). *Inorg. Chim. Acta*, **360**, 1819–1824]. The asymmetric unit contains two complete mol­ecules. Classical hydrogen bonds, as well as C—H⋯O contacts, connect the mol­ecules to infinite chains along the crystallographic *c-*axis direction.

## Abstract 2

The asymmetric unit contains two complete mol­ecules. Classical hydrogen bonds, as well as C—H⋯O contacts, connect the mol­ecules to infinite chains along the *c*-axis direction.

## Graphical abstract

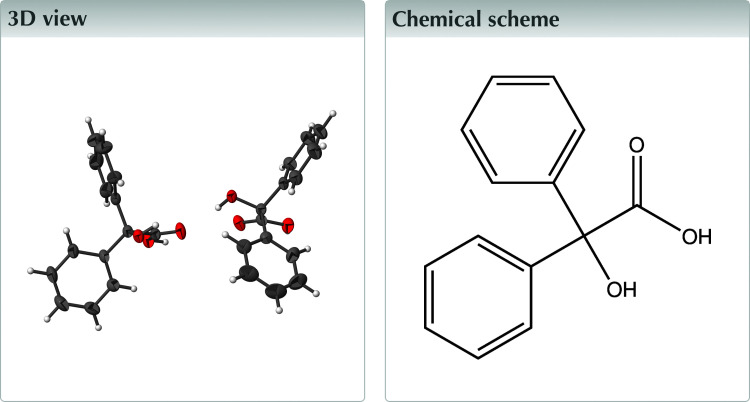

## Structure description

Chelate ligands have found widespread use in coordination chemistry due to the increased stability of coordination compounds they can form in comparison to monodentate ligands (Gade, 1998 ▸). α-Hy­droxy­carb­oxy­lic acids are particularly inter­esting in this aspect as they offer two different donor sites of markedly diverging acidity as potential bonding partners. Upon variation of the substitution pattern on the hydro­carbon backbone, the acidity of the two donor sites can be varied over a wide range, thus turning them into probes for establishing the rules in which p*K*_a_ range coordination to various central atoms of variable Lewis acidity can be observed. In addition, the spatial pretence of the substitution pattern can also be exploited to enable unusual coordination numbers. Furthermore, certain α-hy­droxy­carb­oxy­lic acids form an integral part of metabolic pathways (Berg *et al.*, 2023 ▸), *i.e.* their derivatives might show inter­esting pharmaceutical properties. During an attempt at synthesizing a coordination compound of benzilic acid, the starting material was recovered unchanged, however, diffraction studies found the latter to have crystallized in a monoclinic polymorph. To prevent the waste of valuable measurement time on diffractometers for other researchers the structural details shall be reported herein. The latter is a continuation of our own ongoing inter­est in structural aspects of hy­droxy­carb­oxy­lic acids (Betz & Klüfers, 2007*a* ▸,*b* ▸,*c* ▸,*d* ▸; Betz, Klüfers & Mangstl, 2007 ▸) as well as aromatic carb­oxy­lic acids (Betz, Betzler & Klüfers, 2007 ▸; Betz *et al.*, 2011 ▸; Betz & Gerber, 2011 ▸). The ortho­rhom­bic polymorph of the title compound has been reported earlier (Qiu *et al.*, 2007 ▸) as well as structural data of a number of co-crystallizates of the title compound with, among others, derivatives of pyridine (Ahsan *et al.*, 2023 ▸). Furthermore, the mol­ecular and crystal structures of (*R*)-mandelic acid (Zhang *et al.*, 2013 ▸), (*S*)-mandelic acid (Patil *et al.*, 1987 ▸) as well as racemic mandelic acid (Fischer & Profir, 2003 ▸) and the archaetypical α-hy­droxy­carb­oxy­lic acid – glycolic acid (Pijper, 1971 ▸) – are apparent in the literature.

**Figure 1:**
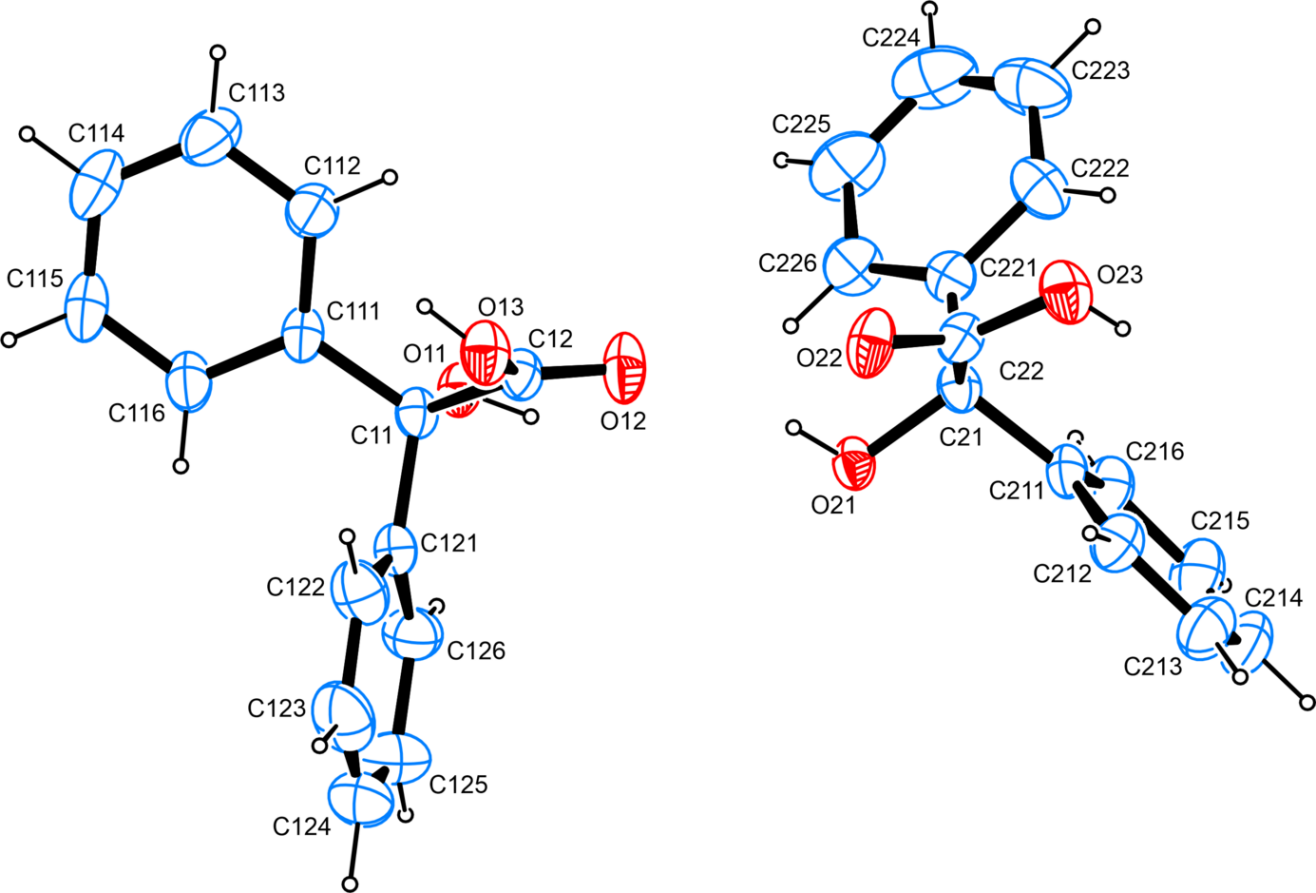
The mol­ecular structure of the title compound, with atom labels and anisotropic displacement ellipsoids (drawn at the 50% probability level).

The title compound is a derivative of hy­droxy­acetic acid bearing two phenyl groups on the carbon scaffold. The asymmetric unit contains two complete mol­ecules (Fig. 1 ▸). The two C=O bond lengths are identical at 1.204 (2) Å, which closely resembles the situation found for the two alcoholic C—O bonds measured at 1.428 (2) Å and 1.431 (2) Å, respectively, in the two independent mol­ecules. The phenyl groups in both mol­ecules are orientated almost perpendicular to one another with the least-squares planes as defined by the respective individual carbon atoms of the aromatic moieties in the two benzilic acid units inter­secting at angles of 83.08 (12) and 85.16 (12)°. The O—C—C—O torsion angles spanning the two protic groups were found at 159.29 (16) and 163.99 (15)°. In comparison, the bond lengths mentioned for the monoclinic polymorph of benzilic acid are found at slightly larger values than the ones reported for the ortho­rhom­bic one while, overall, bond lengths and angles are found in good agreement with other α-hy­droxy­carb­oxy­lic acids whose mol­ecular and crystal structures were determined on grounds of diffraction studies conducted on single crystals and whose metrical parameters have been deposited with the Cambridge Structural Database (Groom *et al.*, 2016 ▸). The structure was refined as a two-component twin with a volume ratio of 73.6:26.4.

**Figure 2:**
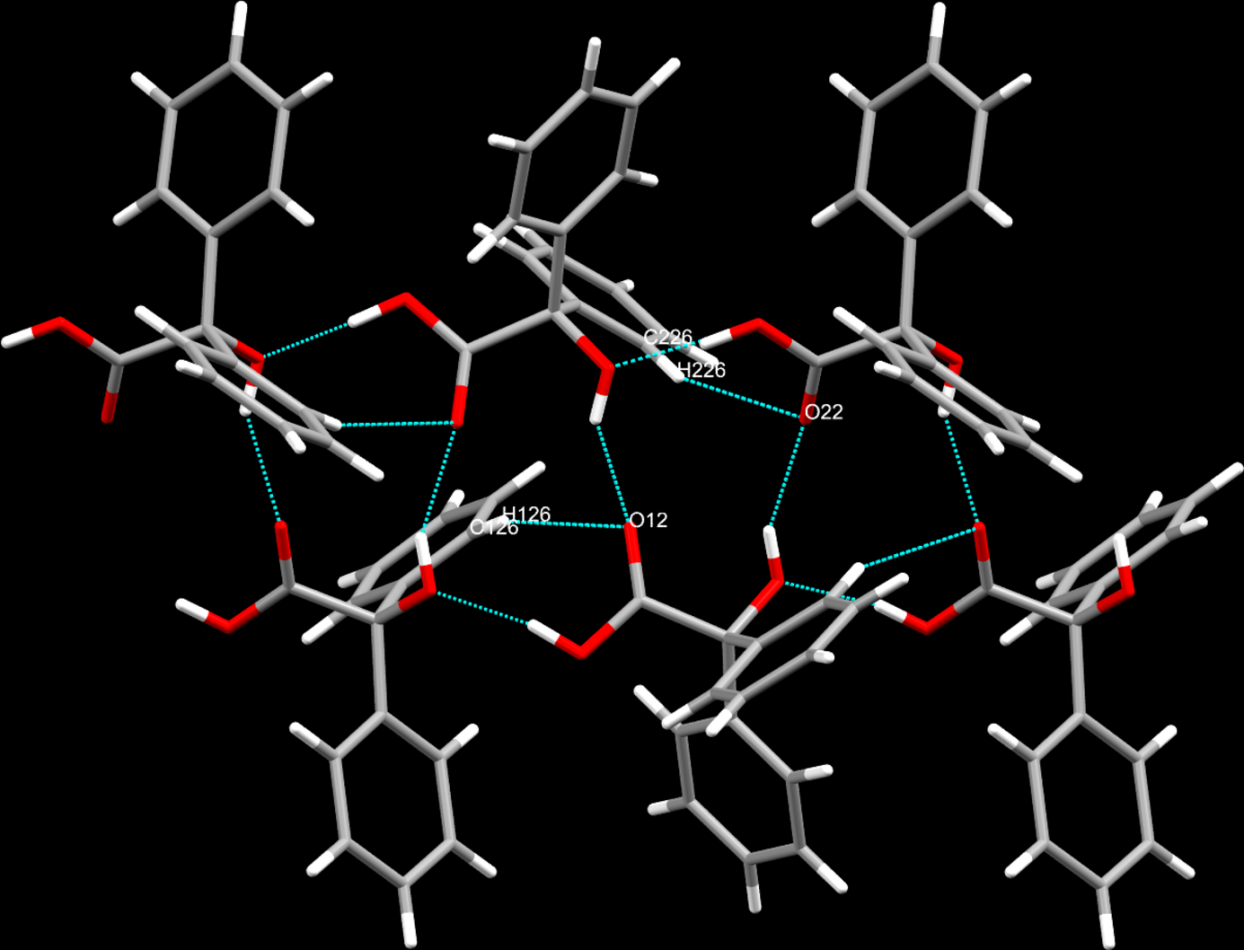
Inter­molecular contacts, view approximately onto the *ac* plane.

In the crystal, classical hydrogen bonds of the O—H⋯O type are found along with C—H⋯O contacts (Table 1 ▸) whose range falls by more than 0.1 Å below the sum of van der Waals radii of the atoms participating in them. While the alcoholic hydroxyl groups invariably form hydrogen bonds to carbonyl-type oxygen atoms as acceptors, the carboxyl-based hydrogen atoms exclusively form hydrogen bonds to the oxygen atoms of the alcoholic groups. It is worthwhile pointing out that the former type of hydrogen bonding alternates in between and connects the two independent mol­ecules present in the asymmetric unit while the latter type of hydrogen bonding described above is fully reserved for each individual of the two independent mol­ecules present in the asymmetric unit as well as its respective symmetry-generated equivalents (Fig. 2 ▸). The C—H⋯O contacts are established by one of the hydrogen atoms in *ortho*-position on one of the phenyl groups and the carbonyl-type oxygen atom of its symmetry-generated equivalent for both independent mol­ecules present in the asymmetric unit. In terms of graph-set analysis (Etter *et al.*, 1990 ▸; Bernstein *et al.*, 1995 ▸), the classical hydrogen bonds require a *DDC^1^_1_(5) 
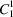
(5)* descriptor on the unary level while the C—H⋯O contacts necessitate a *
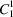
(6) 
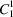
(6)* descriptor on the same level. In total, the mol­ecules are connected to infinite strands along the *c-*axis direction. π-Stacking is not a prominent stabilizing feature in the crystal structure of the title compound with the shortest inter­centroid distance between two aromatic systems measured at 4.5914 (13) Å, apparent in between one of the phenyl groups and its symmetry-generated equivalent.

## Synthesis and crystallization

After an initial unintentional isolation of the crystalline compound from a different synthesis product the compound was targeted by recrystallizing the title compound from THF.

## Refinement

Crystal data, data collection and structure refinement details are summarized in Table 2 ▸.

## Supplementary Material

Crystal structure: contains datablock(s) I. DOI: 10.1107/S2414314624010393/bt4158sup1.cif

Structure factors: contains datablock(s) I. DOI: 10.1107/S2414314624010393/bt4158Isup2.hkl

Supporting information file. DOI: 10.1107/S2414314624010393/bt4158Isup3.cml

CCDC reference: 2393641

Additional supporting information:  crystallographic information; 3D view; checkCIF report

## Figures and Tables

**Table 1:** Hydrogen-bond geometry (Å, °)

*D*—H⋯*A*	*D*—H	H⋯*A*	*D*⋯*A*	*D*—H⋯*A*
O11—H11⋯O22^i^	0.87 (3)	1.98 (3)	2.7776 (19)	153 (3)
O13—H13⋯O11^ii^	0.90 (3)	1.77 (3)	2.6545 (18)	168 (3)
O21—H21⋯O12	0.87 (3)	1.97 (3)	2.7461 (19)	149 (3)
O23—H23⋯O21^ii^	0.93 (4)	1.69 (4)	2.6125 (19)	172 (3)
C126—H126⋯O12^i^	0.95	2.58	3.472 (3)	157
C226—H226⋯O22^i^	0.95	2.55	3.447 (3)	158

Symmetry codes: (i) 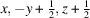; (ii) 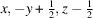.

**Table 2:** Experimental details

Crystal data	
Chemical formula	C_14_H_12_O_3_
*M*_r_	228.24
Crystal system, space group	Monoclinic, *P*2_1_/*c*
Temperature (K)	200
*a*, *b*, *c* (Å)	24.8929 (9), 8.5889 (4), 11.2678 (4)
β (°)	103.0264 (12)
*V* (Å^3^)	2347.09 (16)
*Z*	8
Radiation type	Mo *K*α
μ (mm^−1^)	0.09
Crystal size (mm)	0.39 × 0.15 × 0.05

Data collection	
Diffractometer	Bruker APEXII CCD
Absorption correction	Multi-scan (*SADABS*; Krause *et al.*, 2015 ▸)
*T*_min_, *T*_max_	0.715, 0.746
No. of measured, independent and observed [*I* > 2σ(*I*)] reflections	75829, 5766, 4908
*R*_int_	0.049
(sin θ/λ)_max_ (Å^−1^)	0.666

Refinement	
*R*[*F*^2^ > 2σ(*F*^2^)], *wR*(*F*^2^), *S*	0.081, 0.175, 1.27
No. of reflections	5766
No. of parameters	325
H-atom treatment	H atoms treated by a mixture of independent and constrained refinement
Δρ_max_, Δρ_min_ (e Å^−3^)	0.69, −0.68

Computer programs: *APEX2* and *SAINT* (Bruker, 2014 ▸), *SHELXS97* (Sheldrick 2008 ▸), *ORTEP-3 for Windows* (Farrugia, 2012 ▸), *Mercury* (Macrae *et al.*, 2020 ▸), *SHELXL2019/3* (Sheldrick, 2015 ▸) and *PLATON* (Spek, 2020 ▸).

## full crystallographic data

### 2,2-Diphenyl-2-hydroxyethanoic acid Crystal data

**Table:**

C_14_H_12_O_3_	*F*(000) = 960
*M**_r_* = 228.24	*D*_x_ = 1.292 Mg m^−^^3^
Monoclinic, *P*2_1_/*c*	Mo *K*α radiation, λ = 0.71073 Å
*a* = 24.8929 (9) Å	Cell parameters from 9910 reflections
*b* = 8.5889 (4) Å	θ = 3.0–28.1°
*c* = 11.2678 (4) Å	µ = 0.09 mm^−^^1^
β = 103.0264 (12)°	*T* = 200 K
*V* = 2347.09 (16) Å^3^	Rod, colourless
*Z* = 8	0.39 × 0.15 × 0.05 mm

### 2,2-Diphenyl-2-hydroxyethanoic acid Data collection

**Table:**

Bruker APEXII CCD diffractometer	4908 reflections with *I* > 2σ(*I*)
φ and ω scans	*R*_int_ = 0.049
Absorption correction: multi-scan (SADABS; Krause *et al.*, 2015)	θ_max_ = 28.3°, θ_min_ = 1.9°
*T*_min_ = 0.715, *T*_max_ = 0.746	*h* = −32→31
75829 measured reflections	*k* = −11→11
5766 independent reflections	*l* = −14→15

### 2,2-Diphenyl-2-hydroxyethanoic acid Refinement

**Table:**

Refinement on *F*^2^	Secondary atom site location: difference Fourier map
Least-squares matrix: full	Hydrogen site location: mixed
*R*[*F*^2^ > 2σ(*F*^2^)] = 0.081	H atoms treated by a mixture of independent and constrained refinement
*wR*(*F*^2^) = 0.175	*w* = 1/[σ^2^(*F*_o_^2^) + (0.0977*P*)^2^ + 0.2671*P*] where *P* = (*F*_o_^2^ + 2*F*_c_^2^)/3
*S* = 1.27	(Δ/σ)_max_ = 0.001
5766 reflections	Δρ_max_ = 0.69 e Å^−^^3^
325 parameters	Δρ_min_ = −0.68 e Å^−^^3^
0 restraints	Extinction correction: *SHELXL2019/3* (Sheldrick 2015), Fc^*^=kFc[1+0.001xFc^2^λ^3^/sin(2θ)]^-1/4^
Primary atom site location: structure-invariant direct methods	Extinction coefficient: 0.48 (2)

### 2,2-Diphenyl-2-hydroxyethanoic acid Special details

**Table:**

Geometry. All esds (except the esd in the dihedral angle between two l.s. planes) are estimated using the full covariance matrix. The cell esds are taken into account individually in the estimation of esds in distances, angles and torsion angles; correlations between esds in cell parameters are only used when they are defined by crystal symmetry. An approximate (isotropic) treatment of cell esds is used for estimating esds involving l.s. planes.
Refinement. Refined as a 2-component twin. The aromatic carbon-bound H atoms were placed in calculated positions (C–H 0.95 Å) and were included in the refinement in the riding model approximation, with *U*(H) set to 1.2*U*_eq_(C). The oxygen-bonded H atoms were located on a DFM and refined freely.

### 2,2-Diphenyl-2-hydroxyethanoic acid Fractional atomic coordinates and isotropic or equivalent isotropic displacement parameters (Å^2^)

**Table:**

	*x*	*y*	*z*	*U*_iso_*/*U*_eq_	
O11	0.82588 (5)	0.28942 (15)	0.85279 (12)	0.0277 (3)	
H11	0.7908 (11)	0.280 (3)	0.821 (3)	0.043 (7)*	
O12	0.78554 (6)	0.2500 (2)	0.61874 (14)	0.0413 (4)	
O13	0.86377 (6)	0.14787 (19)	0.58744 (12)	0.0363 (4)	
H13	0.8461 (13)	0.167 (3)	0.510 (3)	0.059 (8)*	
O21	0.67403 (5)	0.21606 (16)	0.52974 (12)	0.0285 (3)	
H21	0.7089 (12)	0.218 (4)	0.529 (3)	0.055 (8)*	
O22	0.71287 (5)	0.2323 (2)	0.33290 (13)	0.0386 (4)	
O23	0.63800 (6)	0.35107 (19)	0.22422 (13)	0.0382 (4)	
H23	0.6527 (15)	0.335 (4)	0.156 (4)	0.075 (10)*	
C11	0.85555 (7)	0.1825 (2)	0.79411 (15)	0.0254 (4)	
C12	0.83115 (7)	0.1979 (2)	0.65616 (16)	0.0287 (4)	
C21	0.64460 (7)	0.3188 (2)	0.43824 (16)	0.0263 (4)	
C22	0.66896 (7)	0.2952 (2)	0.32526 (16)	0.0283 (4)	
C111	0.91561 (7)	0.2350 (2)	0.83067 (17)	0.0286 (4)	
C112	0.93433 (8)	0.3575 (2)	0.77093 (19)	0.0360 (5)	
H112	0.910675	0.402087	0.701161	0.043*	
C113	0.98732 (9)	0.4159 (3)	0.8120 (2)	0.0466 (5)	
H113	0.999907	0.498960	0.769556	0.056*	
C114	1.02166 (9)	0.3535 (3)	0.9142 (2)	0.0481 (6)	
H114	1.057827	0.393711	0.942561	0.058*	
C115	1.00331 (8)	0.2325 (3)	0.9750 (2)	0.0457 (6)	
H115	1.026914	0.189565	1.045502	0.055*	
C116	0.95052 (8)	0.1730 (3)	0.93373 (19)	0.0369 (5)	
H116	0.938210	0.089506	0.976152	0.044*	
C121	0.84577 (7)	0.0136 (2)	0.82826 (17)	0.0296 (4)	
C122	0.86857 (10)	−0.1088 (3)	0.7756 (2)	0.0437 (5)	
H122	0.890989	−0.087974	0.719498	0.052*	
C123	0.85863 (11)	−0.2614 (3)	0.8050 (3)	0.0580 (7)	
H123	0.874237	−0.344495	0.768470	0.070*	
C124	0.82638 (12)	−0.2937 (3)	0.8866 (3)	0.0590 (7)	
H124	0.819454	−0.398552	0.905504	0.071*	
C125	0.80435 (12)	−0.1735 (3)	0.9403 (3)	0.0565 (7)	
H125	0.782690	−0.195391	0.997718	0.068*	
C126	0.81351 (9)	−0.0196 (2)	0.9112 (2)	0.0412 (5)	
H126	0.797695	0.062824	0.948027	0.049*	
C211	0.58455 (7)	0.2658 (2)	0.41614 (17)	0.0284 (4)	
C212	0.56538 (9)	0.1445 (2)	0.33656 (19)	0.0369 (5)	
H212	0.588665	0.101099	0.288964	0.044*	
C213	0.51266 (10)	0.0860 (3)	0.3258 (2)	0.0471 (6)	
H213	0.499914	0.003189	0.270786	0.057*	
C214	0.47850 (9)	0.1482 (3)	0.3951 (2)	0.0476 (6)	
H214	0.442358	0.108030	0.387924	0.057*	
C215	0.49708 (8)	0.2683 (3)	0.4743 (2)	0.0474 (6)	
H215	0.473752	0.310708	0.522167	0.057*	
C216	0.55002 (8)	0.3284 (3)	0.48470 (19)	0.0377 (5)	
H216	0.562429	0.412396	0.538834	0.045*	
C221	0.65375 (8)	0.4897 (2)	0.47835 (17)	0.0298 (4)	
C222	0.63148 (10)	0.6090 (3)	0.3992 (2)	0.0456 (5)	
H222	0.610286	0.584967	0.320101	0.055*	
C223	0.64013 (11)	0.7627 (3)	0.4353 (3)	0.0600 (7)	
H223	0.625195	0.843777	0.380342	0.072*	
C224	0.67020 (12)	0.7993 (3)	0.5503 (3)	0.0623 (8)	
H224	0.676068	0.905062	0.574489	0.075*	
C225	0.69157 (12)	0.6821 (3)	0.6294 (3)	0.0576 (7)	
H225	0.711717	0.706791	0.709236	0.069*	
C226	0.68395 (9)	0.5271 (2)	0.5935 (2)	0.0407 (5)	
H226	0.699573	0.446578	0.648316	0.049*	

### 2,2-Diphenyl-2-hydroxyethanoic acid Atomic displacement parameters (Å^2^)

**Table:**

	*U*^11^	*U*^22^	*U*^33^	*U*^12^	*U*^13^	*U*^23^
O11	0.0226 (7)	0.0369 (7)	0.0237 (6)	0.0034 (5)	0.0058 (5)	−0.0011 (5)
O12	0.0254 (7)	0.0705 (10)	0.0260 (7)	0.0067 (7)	0.0013 (6)	0.0049 (7)
O13	0.0355 (8)	0.0526 (9)	0.0208 (6)	0.0068 (6)	0.0061 (6)	−0.0005 (6)
O21	0.0243 (7)	0.0383 (7)	0.0225 (6)	0.0033 (5)	0.0044 (5)	0.0039 (5)
O22	0.0246 (7)	0.0634 (9)	0.0285 (7)	0.0018 (6)	0.0074 (6)	−0.0072 (6)
O23	0.0411 (8)	0.0529 (9)	0.0216 (7)	0.0073 (6)	0.0092 (6)	0.0041 (6)
C11	0.0235 (8)	0.0336 (9)	0.0193 (8)	0.0030 (6)	0.0053 (6)	0.0002 (6)
C12	0.0263 (8)	0.0373 (9)	0.0220 (8)	0.0000 (7)	0.0044 (7)	0.0022 (7)
C21	0.0270 (9)	0.0326 (8)	0.0192 (8)	0.0026 (7)	0.0048 (7)	0.0011 (6)
C22	0.0259 (9)	0.0365 (9)	0.0230 (8)	−0.0034 (7)	0.0066 (7)	−0.0024 (7)
C111	0.0221 (8)	0.0398 (9)	0.0232 (9)	0.0029 (7)	0.0038 (7)	−0.0008 (7)
C112	0.0332 (10)	0.0439 (11)	0.0310 (10)	−0.0025 (8)	0.0075 (8)	0.0018 (8)
C113	0.0393 (11)	0.0561 (13)	0.0460 (12)	−0.0140 (10)	0.0132 (10)	−0.0008 (10)
C114	0.0273 (10)	0.0677 (15)	0.0484 (13)	−0.0075 (9)	0.0061 (9)	−0.0101 (11)
C115	0.0285 (10)	0.0620 (14)	0.0402 (12)	0.0029 (9)	−0.0059 (10)	−0.0006 (10)
C116	0.0291 (9)	0.0486 (11)	0.0305 (10)	0.0029 (8)	0.0014 (8)	0.0042 (8)
C121	0.0268 (9)	0.0336 (9)	0.0258 (8)	0.0024 (7)	0.0005 (7)	0.0014 (7)
C122	0.0475 (13)	0.0402 (11)	0.0424 (12)	0.0110 (9)	0.0079 (10)	−0.0022 (9)
C123	0.0641 (16)	0.0369 (11)	0.0652 (17)	0.0135 (11)	−0.0020 (15)	−0.0066 (11)
C124	0.0639 (16)	0.0333 (11)	0.0723 (18)	−0.0043 (10)	−0.0001 (14)	0.0082 (11)
C125	0.0618 (16)	0.0465 (12)	0.0638 (17)	−0.0089 (11)	0.0195 (14)	0.0140 (12)
C126	0.0445 (12)	0.0381 (10)	0.0427 (11)	−0.0022 (8)	0.0134 (10)	0.0043 (9)
C211	0.0232 (8)	0.0383 (9)	0.0223 (9)	0.0027 (7)	0.0025 (7)	0.0022 (7)
C212	0.0348 (10)	0.0442 (11)	0.0321 (10)	−0.0031 (8)	0.0082 (8)	−0.0041 (8)
C213	0.0412 (12)	0.0574 (14)	0.0404 (11)	−0.0112 (10)	0.0040 (9)	−0.0046 (10)
C214	0.0287 (10)	0.0669 (15)	0.0461 (13)	−0.0078 (10)	0.0064 (9)	0.0037 (11)
C215	0.0291 (10)	0.0683 (15)	0.0473 (14)	0.0022 (10)	0.0139 (10)	−0.0017 (11)
C216	0.0305 (10)	0.0485 (11)	0.0357 (10)	0.0028 (8)	0.0109 (8)	−0.0019 (8)
C221	0.0282 (9)	0.0319 (9)	0.0315 (9)	0.0028 (7)	0.0117 (7)	−0.0015 (7)
C222	0.0476 (13)	0.0412 (11)	0.0481 (13)	0.0126 (9)	0.0111 (10)	0.0070 (9)
C223	0.0700 (17)	0.0368 (11)	0.080 (2)	0.0129 (12)	0.0306 (17)	0.0089 (12)
C224	0.0674 (17)	0.0362 (12)	0.092 (2)	−0.0025 (11)	0.0373 (17)	−0.0166 (13)
C225	0.0621 (16)	0.0484 (13)	0.0635 (17)	−0.0087 (11)	0.0167 (13)	−0.0231 (12)
C226	0.0472 (12)	0.0388 (10)	0.0354 (10)	−0.0004 (8)	0.0081 (9)	−0.0078 (8)

### 2,2-Diphenyl-2-hydroxyethanoic acid Geometric parameters (Å, º)

**Table:**

O11—C11	1.431 (2)	C122—H122	0.9500
O11—H11	0.87 (3)	C123—C124	1.378 (5)
O12—C12	1.204 (2)	C123—H123	0.9500
O13—C12	1.314 (2)	C124—C125	1.372 (4)
O13—H13	0.90 (3)	C124—H124	0.9500
O21—C21	1.428 (2)	C125—C126	1.393 (3)
O21—H21	0.87 (3)	C125—H125	0.9500
O22—C22	1.204 (2)	C126—H126	0.9500
O23—C22	1.314 (2)	C211—C212	1.387 (3)
O23—H23	0.93 (4)	C211—C216	1.387 (3)
C11—C111	1.527 (2)	C212—C213	1.385 (3)
C11—C121	1.534 (2)	C212—H212	0.9500
C11—C12	1.542 (2)	C213—C214	1.385 (4)
C21—C211	1.528 (2)	C213—H213	0.9500
C21—C221	1.538 (2)	C214—C215	1.374 (4)
C21—C22	1.542 (2)	C214—H214	0.9500
C111—C112	1.385 (3)	C215—C216	1.395 (3)
C111—C116	1.390 (3)	C215—H215	0.9500
C112—C113	1.390 (3)	C216—H216	0.9500
C112—H112	0.9500	C221—C226	1.382 (3)
C113—C114	1.379 (4)	C221—C222	1.389 (3)
C113—H113	0.9500	C222—C223	1.384 (4)
C114—C115	1.378 (4)	C222—H222	0.9500
C114—H114	0.9500	C223—C224	1.379 (5)
C115—C116	1.389 (3)	C223—H223	0.9500
C115—H115	0.9500	C224—C225	1.370 (4)
C116—H116	0.9500	C224—H224	0.9500
C121—C122	1.390 (3)	C225—C226	1.392 (3)
C121—C126	1.392 (3)	C225—H225	0.9500
C122—C123	1.388 (4)	C226—H226	0.9500
			
C11—O11—H11	109.0 (18)	C124—C123—H123	119.6
C12—O13—H13	106.1 (19)	C122—C123—H123	119.6
C21—O21—H21	109 (2)	C125—C124—C123	119.5 (2)
C22—O23—H23	113 (2)	C125—C124—H124	120.2
O11—C11—C111	105.17 (14)	C123—C124—H124	120.2
O11—C11—C121	111.31 (14)	C124—C125—C126	120.5 (3)
C111—C11—C121	114.39 (15)	C124—C125—H125	119.8
O11—C11—C12	106.46 (13)	C126—C125—H125	119.8
C111—C11—C12	112.70 (15)	C121—C126—C125	120.2 (2)
C121—C11—C12	106.60 (14)	C121—C126—H126	119.9
O12—C12—O13	125.03 (18)	C125—C126—H126	119.9
O12—C12—C11	120.73 (17)	C212—C211—C216	119.03 (18)
O13—C12—C11	114.23 (15)	C212—C211—C21	120.68 (17)
O21—C21—C211	104.93 (14)	C216—C211—C21	119.99 (17)
O21—C21—C221	111.09 (14)	C213—C212—C211	120.7 (2)
C211—C21—C221	114.00 (15)	C213—C212—H212	119.7
O21—C21—C22	106.35 (14)	C211—C212—H212	119.7
C211—C21—C22	112.25 (15)	C212—C213—C214	120.1 (2)
C221—C21—C22	107.97 (14)	C212—C213—H213	120.0
O22—C22—O23	124.86 (17)	C214—C213—H213	120.0
O22—C22—C21	121.29 (17)	C215—C214—C213	119.7 (2)
O23—C22—C21	113.85 (15)	C215—C214—H214	120.1
C112—C111—C116	118.79 (18)	C213—C214—H214	120.1
C112—C111—C11	120.63 (17)	C214—C215—C216	120.4 (2)
C116—C111—C11	120.13 (18)	C214—C215—H215	119.8
C111—C112—C113	120.7 (2)	C216—C215—H215	119.8
C111—C112—H112	119.6	C211—C216—C215	120.1 (2)
C113—C112—H112	119.6	C211—C216—H216	120.0
C114—C113—C112	120.1 (2)	C215—C216—H216	120.0
C114—C113—H113	120.0	C226—C221—C222	119.06 (19)
C112—C113—H113	120.0	C226—C221—C21	120.69 (17)
C115—C114—C113	119.7 (2)	C222—C221—C21	120.25 (18)
C115—C114—H114	120.1	C223—C222—C221	120.1 (2)
C113—C114—H114	120.1	C223—C222—H222	120.0
C114—C115—C116	120.4 (2)	C221—C222—H222	120.0
C114—C115—H115	119.8	C224—C223—C222	120.6 (3)
C116—C115—H115	119.8	C224—C223—H223	119.7
C115—C116—C111	120.3 (2)	C222—C223—H223	119.7
C115—C116—H116	119.8	C225—C224—C223	119.5 (2)
C111—C116—H116	119.8	C225—C224—H224	120.2
C122—C121—C126	118.96 (19)	C223—C224—H224	120.2
C122—C121—C11	120.33 (18)	C224—C225—C226	120.4 (3)
C126—C121—C11	120.71 (17)	C224—C225—H225	119.8
C123—C122—C121	120.0 (2)	C226—C225—H225	119.8
C123—C122—H122	120.0	C221—C226—C225	120.3 (2)
C121—C122—H122	120.0	C221—C226—H226	119.9
C124—C123—C122	120.8 (3)	C225—C226—H226	119.9
			
O11—C11—C12—O12	−21.8 (2)	C121—C122—C123—C124	−0.2 (4)
C111—C11—C12—O12	−136.58 (19)	C122—C123—C124—C125	−0.7 (4)
C121—C11—C12—O12	97.1 (2)	C123—C124—C125—C126	1.2 (4)
O11—C11—C12—O13	159.29 (16)	C122—C121—C126—C125	−0.1 (3)
C111—C11—C12—O13	44.5 (2)	C11—C121—C126—C125	179.2 (2)
C121—C11—C12—O13	−81.80 (19)	C124—C125—C126—C121	−0.8 (4)
O21—C21—C22—O22	−16.9 (2)	O21—C21—C211—C212	−84.3 (2)
C211—C21—C22—O22	−131.11 (18)	C221—C21—C211—C212	153.97 (17)
C221—C21—C22—O22	102.4 (2)	C22—C21—C211—C212	30.8 (2)
O21—C21—C22—O23	163.99 (15)	O21—C21—C211—C216	89.3 (2)
C211—C21—C22—O23	49.8 (2)	C221—C21—C211—C216	−32.4 (2)
C221—C21—C22—O23	−76.71 (19)	C22—C21—C211—C216	−155.58 (18)
O11—C11—C111—C112	−80.9 (2)	C216—C211—C212—C213	−0.3 (3)
C121—C11—C111—C112	156.67 (18)	C21—C211—C212—C213	173.42 (19)
C12—C11—C111—C112	34.7 (2)	C211—C212—C213—C214	−0.2 (3)
O11—C11—C111—C116	91.3 (2)	C212—C213—C214—C215	0.2 (4)
C121—C11—C111—C116	−31.1 (2)	C213—C214—C215—C216	0.3 (4)
C12—C11—C111—C116	−153.11 (18)	C212—C211—C216—C215	0.8 (3)
C116—C111—C112—C113	1.0 (3)	C21—C211—C216—C215	−173.0 (2)
C11—C111—C112—C113	173.34 (19)	C214—C215—C216—C211	−0.8 (4)
C111—C112—C113—C114	−0.9 (4)	O21—C21—C221—C226	−4.3 (2)
C112—C113—C114—C115	0.3 (4)	C211—C21—C221—C226	114.0 (2)
C113—C114—C115—C116	0.1 (4)	C22—C21—C221—C226	−120.55 (19)
C114—C115—C116—C111	0.0 (4)	O21—C21—C221—C222	175.96 (18)
C112—C111—C116—C115	−0.5 (3)	C211—C21—C221—C222	−65.7 (2)
C11—C111—C116—C115	−172.9 (2)	C22—C21—C221—C222	59.7 (2)
O11—C11—C121—C122	176.53 (17)	C226—C221—C222—C223	0.7 (3)
C111—C11—C121—C122	−64.4 (2)	C21—C221—C222—C223	−179.6 (2)
C12—C11—C121—C122	60.8 (2)	C221—C222—C223—C224	−0.8 (4)
O11—C11—C121—C126	−2.8 (2)	C222—C223—C224—C225	−0.1 (4)
C111—C11—C121—C126	116.3 (2)	C223—C224—C225—C226	1.2 (4)
C12—C11—C121—C126	−118.46 (19)	C222—C221—C226—C225	0.4 (3)
C126—C121—C122—C123	0.6 (3)	C21—C221—C226—C225	−179.4 (2)
C11—C121—C122—C123	−178.67 (19)	C224—C225—C226—C221	−1.3 (4)

### 2,2-Diphenyl-2-hydroxyethanoic acid Hydrogen-bond geometry (Å, º)

**Table:**

*D*—H···*A*	*D*—H	H···*A*	*D*···*A*	*D*—H···*A*
O11—H11···O22^i^	0.87 (3)	1.98 (3)	2.7776 (19)	153 (3)
O13—H13···O11^ii^	0.90 (3)	1.77 (3)	2.6545 (18)	168 (3)
O21—H21···O12	0.87 (3)	1.97 (3)	2.7461 (19)	149 (3)
O23—H23···O21^ii^	0.93 (4)	1.69 (4)	2.6125 (19)	172 (3)
C126—H126···O12^i^	0.95	2.58	3.472 (3)	157
C226—H226···O22^i^	0.95	2.55	3.447 (3)	158

Symmetry codes: (i) *x*, −*y*+1/2, *z*+1/2; (ii) *x*, −*y*+1/2, *z*−1/2.
